# “Pharmacogenetics of Cancer” - *Cancer Drug Resistance* special issue

**DOI:** 10.20517/cdr.2020.10

**Published:** 2020-02-20

**Authors:** Enrico Mini, Stefania Nobili

**Affiliations:** ^1^Department of Health Sciences, Section of Clinical Pharmacology and Oncology, University of Florence, Florence 50139, Italy.; ^2^Cancer Pharmacology Working Group of the Italian Society of Pharmacology, Milan 20129, Italy.

Pharmacogenetics and pharmacogenomics have expanded rapidly over the past years and represent one of the bases of precision medicine. The application of pharmacogenetic and pharmacogenomic approaches and tools for the selection of medications and their dosage to improve patient care is nowadays substantially increased^[1,2]^. Prescription drug information sheets in several therapeutic areas (psychiatric, cardiovascular, analgesic, antiviral, and anticancer drugs) provide current information on pharmacogenetic and pharmacogenomic biomarkers for over 200 medicines including mandatory or recommended biomarker testing prior to initiating therapy^[3]^.

The pharmacological treatment of cancer is an ideal field for the application of pharmacogenetics and pharmacogenomics in clinical practice. Substantial advances in pharmacological therapy of cancer over the past three decades have been made through the discovery of cancer molecular hallmarks and the development of specific anticancer therapeutics [e.g., chemical protein kinase inhibitors, anti-membrane receptor monoclonal antibodies (MoAbs), and more recently transformative cell and gene therapeutics]. These drugs target tumor cells, immune cells, and other cells of the cancer microenvironment and may be selected on the basis of the presence of specific molecular alterations including somatic or germline mutations^[4]^.

However, anticancer drug toxicity remains a major issue in cancer care. This is typically associated with the use of classical cytotoxic chemotherapeutic agents, often leading to considerable morbidity and mortality, but is also commonly associated with the use of targeted cancer therapeutics, with significant adverse event profiles from both on and off target effects^[5]^.

Moreover, intrinsic or acquired drug resistance remains a major biological and clinical phenomenon leading to anticancer treatment failure^[6]^.

Testing of cancer patients for genetic markers of efficacy or toxicity of anticancer therapeutics is increasingly being used as a result of clinical studies based on genotype stratification, availability of approved clinical tests and companion diagnostic biomarkers, and the continually more robust clinical pharmacogenetic and pharmacogenomic information and guidelines^[7-9]^.

This special issue of *Cancer Drug Resistance* is designed to provide an overview on the role of pharmacogenetics and pharmacogenomics in the guidance of cancer treatment prescription; on the current strategies of pharmacogenetic and pharmacogenomic discovery and their implementation in clinical cancer drug development; and on the available information on drug labels, web resources, and guidelines, with particular regard to the knowledge of germline variations for specific cancer drug classes and their relevance to increased risks of adverse effects or decreased therapeutic efficacy [e.g., fluoropyrimidines, camptothecins, thiopurines, asparaginase, immune checkpoint inhibitors and other monoclonal antibodies, and poly-adenosyl-ribose polymerase (PARP) inhibitors]. Finally, the integration of somatic pharmacogenomic markers into clinical practice with examples of some relevant cancer types such as breast cancer, gastrointestinal stromal tumor (GIST), and cholangiocarcinoma, as well as an overview on their role in the development of cancer drug resistance, is also addressed.

The article by Kaehler and Cascorbi^[10]^ focuses on a relevant distinction in cancer pharmacogenetics. Cancer pharmacogenetics and pharmacogenomics imply in fact a complex conundrum of germline variants from normal cells and somatic mutations from tumor cells. As aforesaid, somatic mutations represent important druggable targets or biomarkers, but germline variants potentially predict adverse drug effects or drug response. The authors evaluated the relevance of hereditary variants in absorption, distribution, metabolism, excretion (ADME) of enzymes/proteins [such as thiopurine *S*-methyltransferase (TPMT), UDP-glucuronosyltransferase (UGT1A1), and dihydropyrimidine dehydrogenase (DPD)] in predicting genetic adverse events as well as drug transporters (ABCB1, ABCG2, and ABCC subfamilies of ATP binding cassette transporters) and target enzymes in predicting drug efficacy with respect to both cytotoxic agents and targeted agents.

They critically discussed gene expression regulation with regards to epigenetics and post-transcriptional modification. They also addressed the concept that regulation of genes involved in the metabolism and site of action of anticancer agents may be modulated by epigenetics (e.g., DNA methylation or histone modification) at the post-transcriptional level.

Olivera *et al.*^[11]^ provided an overview of germline pharmacogenetics currently applied to the clinical practice in oncology. On this basis, the authors described the state-of-the-art for several drugs in relation to specific polymorphisms, as reported in pivotal tools useful for therapeutic decisions. In particular, they mainly referred to information reported on the PharmGKB website with particular focus on the drug/polymorphism relationships based on the levels of evidence reported in the PharmGKB Clinical Annotations, genetic information reported on drug labels of main drug regulatory agencies, and guidelines elaborated by international expert consortia (e.g., Clinical Pharmacogenetics Implementation Consortium and Dutch Pharmacogenetics Working Group). On this basis, recommendations for the administration of thiopurines in relation to genetic polymorphisms of *TPMT*, fluoropyrimidines in relation to those of dihydropyrimidine dehydrogenase (*DPYD*), and drugs substrate of cytochrome P450 family members whose polymorphisms have been associated with drug response (i.e., efficacy and toxicity) are described.

The review by Crisafulli *et al.*^[12]^ deals with the several strategies currently available to study germline polymorphisms, tumor biomarkers, cancer drivers, and actionable targets for cancer treatment, in relation to specific endpoints of response related to efficacy and/or toxicity. Researchers are today called to plan the optimal identification strategy (e.g., candidate genes and whole genome analysis) based on the aims of their studies. In particular, the availability of several next generation sequencing technologies allows the discovery of novel cancer-driving mutations as well as druggable mutations. Evidence found using these approaches has to undergo validation processes before their application in the clinical practice as diagnostic or prognostic biomarkers. Thus, regulatory agencies have established recommendations to adopt pharmacogenetic and pharmacogenomic methods in research and diagnostics.

Pharmacogenetics and pharmacogenomics may also successfully drive the discovery and development process of new anticancer agents by greatly reducing the attrition rate issue, as reported in the review by Tarantino *et al.*^[13]^. A strategy to decrease this occurrence is represented, for instance, by alternative trial designs such as the basket and umbrella trials, in which the histology-oriented approach is replaced by the molecular alteration-centered ones^[14,15]^. The introduction of pharmacogenetics/pharmacogenomics principles in the early drug developmental phases may also contribute to the reduction of the high attrition rates, although not every early phase of clinical trials benefits to the same extent from pharmacogenetics/pharmacogenomics implementation. The more promising phase of clinical trials for identifying efficacy biomarkers is probably represented by phase II studies due to the possibility to include biomarkers in the trial design and to randomize patients. Liquid biopsy, which is today routinely used to detect specific somatic mutations predictive of anticancer drug efficacy/resistance, may also be implemented in biomarker-driven clinical trials in order to avoid multiple traditional biopsies that should be performed longitudinally. All these opportunities have the potential to make easier and faster translation of findings from clinical trials into clinical practice benefits^[16]^.

Fluoropyrimidines (i.e., 5-fluorouracil and capecitabine) that still represent a backbone in the treatment of several solid tumors, including colorectal cancer, have been widely investigated from a pharmacogenetic point of view since polymorphisms of genes that codify for enzymes involved in their complex metabolism and mechanism of action have been related to their adverse events and, to a lesser extent, their efficacy. The review by De Mattia *et al.*^[17]^ points out the current recommendations on the use of genetic tests for the detection of deleterious variants in DPYD. These authors also provided information based on their previously published data on the higher economic burden of toxicity management for colorectal cancer patients harboring *DPYD* polymorphisms and treated with fluoropyrimidines compared with wild-type patients. Polymorphisms of two major pharmacogenes, namely thymidylate synthase (*TYMS*) and 5,10-methylentetrahydrofolate reductase (*MTHFR*), which are involved in the mechanism of action of fluoropyrimidines, have been suggested in the last decades to have a role not only in their efficacy but also in their toxicity. The authors critically reviewed the available evidence on these aspects. Although genetic variants of various other activating or inactivating metabolism enzymes of fluoropyrimidines have been suggested to play a role in fluoropyrimidine toxicity (e.g., uridine monophosphate synthase, cytidine deaminase, and carboxylesterase 1 and 2), as have immune-related genes [e.g., human leukocyte antigen (HLA)], the authors concluded that, overall, only loss of function *DPYD* polymorphisms represent recognized pharmacogenetic markers with statistical and clinical significance for the prediction of fluoropyrimidine-induced toxicity^[18]^.

The review of Franca *et al.*^[19]^ tackles similar issues on the use of another class of antimetabolite drugs. Thiopurines include mercaptopurine and thioguanine, which are active in several forms of leukemia including acute lymphoblastic leukemia (ALL), and azathioprine, which is used as immunosuppressive agent in the treatment of autoimmune diseases and in regimens to prevent transplant rejection. Catabolism of thiopurines is mediated by xanthine oxidase and TPMT, being the latter responsible for their main inactivation pathway. The wide use of mercaptopurine in pediatric ALL in combination with other anticancer drugs, along with the established role of TPMT genetic variants in mercaptopurine-related toxicities, has led through the years to the definition of national and international guidelines that are nowadays commonly followed^[20]^. More recently, clinical guidelines are also available for nudix hydrolase 15 (NUDT15) polymorphisms^[20]^. This is a ubiquitously expressed nucleotide triphosphate diphosphatase enzyme that inactivates triphosphate thionucleotides. In patients carrying deleterious *NUDT15* polymorphisms, increased toxic levels of thionucleotides are achieved after mercaptopurine treatment. Other enzymes, such as inosine triphosphate pyrophosphatase that catalyzes the hydrolysis of the triphosphate moieties from noncanonical (deoxy-) purine triphosphate, protein kinase C, and casein kinase substrate in neurons protein 2 (PACSIN2), a protein close to membranes, have been suggested to play a role in thiopurine adverse events but further investigation is required.

By keeping the focus on hemolymphoid cancers, Abaji and Krajinovic^[21]^ discussed the pharmacogenetics of asparaginase, a drug used in these diseases, in particular in ALL. Although its mechanism of action is still not fully clarified, the main evidence suggests its involvement in the hydrolysis of asparagine and glutamine in serum. Due to the xenobiotic nature of asparaginase, hypersensitivity reactions represent a quite common adverse event, counteracted to a relevant extent by premedication treatment. From GWAS studies, genes whose polymorphisms could play a role in this toxic event have emerged [e.g., polypeptide N-acetylgalactosaminyltransferase 10 (*GALNT10*)]. In addition, polymorphisms in *HLA* Class II region alleles have been associated with hypersensitivity related to asparaginase. For other adverse events, such as pancreatitis or thrombosis, genetic polymorphisms identified by candidate gene or GWAS approaches have been reported [e.g., unc-51-like autophagy activating kinase (*ULK2*) and nuclear factor of activated T cells 2 (*NFATC2*)]. Correlations between asparaginase efficacy and genetic polymorphisms have also been described [e.g., asparagine synthetase (*ASNS*) activating transcription factor 5 (*ATF5*) and argininosuccinate synthase 1 (*ASS1*)]. However, relationships between asparaginase toxicity/efficacy and germline polymorphisms have still not been validated; thus, guidelines on this associations are lacking.

Differently from cytotoxic anticancer drugs, most of the evidence for pharmacogenetics of targeted agents refers to efficacy more than toxicity and commonly to tumor genome more than host genome. This is the case of MoAbs, including immune checkpoint inhibitors, as reviewed by Shek *et al.*^[22]^. Polymorphisms in genes codifying target proteins or protein substrates in the signaling pathways of MoAbs (e.g., vascular endothelial growth factor and RAS) as well as polymorphisms in genes codifying proteins involved in ADME of MoAbs have been shown to play a role in their efficacy [e.g., Fc fragment of IgG receptor IIIa (*FCGR3A*) gene polymorphisms]. More recently, polymorphisms in immune checkpoint genes [e.g., cytotoxic T-lymphocyte associated protein 4 (*CTLA4*), CD274 molecule, and *PD-L1*] have also been linked to the efficacy of immune checkpoints inhibitors. The presence of such polymorphisms impairs or strongly decreases the response to MoAbs. This field of cancer therapeutics, which is probably the most promising, could greatly benefit from the implementation of next generation sequencing approaches in clinical practice prior to treatment initiation.

The review of Murthy and Muggia^[23]^ describes clinical trials that have led to the approval of PARP inhibitors in *BRCA*-mutated ovarian and breast cancers (to which pancreatic cancer was added in December 2019), pharmacological differences between the various PARP inhibitors, and the emerging mechanisms of resistance. The authors focused on the targets of these drugs (mainly, PARP1 and PARP2 but also PARP1-3 for some of them) as well as on the pharmacological role. This review provides an overview for future development of these drugs. Due to the frequent occurrence of tumor drug resistance, mainly due to restoration of homologous recombination, replication fork dynamics, PARylation balance, loss of PARP1, and drug efflux, the study of biomarkers predictive of this phenomenon is pivotal, as is the identification of their most active schedule (e.g., monotherapy *vs.* combination therapy) or sequence of treatment.

As mentioned above, the occurrence of intrinsic or acquired drug resistance represents a major reason for anticancer treatment failure. Kumar *et al.*^[24]^ discussed traditional and more recently emerged mechanisms of tumor drug resistance, thus providing an overview of this complex phenomenon. In addition to classical mechanisms represented mainly by efflux pumps or detoxifying enzymes, other key processes involved in drug resistance (e.g., tumor heterogeneity, reactivation of drug targets, hyperactivation of alternative pathways, cross-talk with the microenvironment, altered DNA response and DNA repair, epigenetic alterations, impairment in apoptosis/autophagy, and presence of cancer stem cells) have been widely investigated in recent years. Examples are represented by the inactivation of transcription factors such as Forkhead box proteins by the hyperactivation of PI3K/Akt signaling and the protective role of focal adhesion kinase (FAK alias PTK2) through induction of NF-κB pathway mediated cytokine production in response to DNA damage. Due to the complexity of tumor drug resistance, the concurrent targeting of multiple mechanisms responsible for this phenomenon could represent the optimal strategy.

Concerning the development of tumor drug resistance, intratumoral heterogeneity represents a critical factor. Breast cancer, the most frequent neoplasms worldwide, is a highly heterogenous disease from the molecular point of view and this directly impacts response to drug treatment. Belizario and Loggulo^[25]^ reviewed the state-of-the-art of the current knowledge on breast cancer molecular subtypes and their relationships with prognosis and treatment response. In addition to the already acquired findings, the current availability of the newest biomolecular technologies combined with computational integrative methods will obtain an increasingly accurate prediction of treatment response by interrogating specific pivotal areas such as tumor and peritumoral microenvironments and by identifying new biomarkers useful to further stratify patients and ameliorate personalized treatment.

Similar considerations may be applied to the heterogeneity of GIST, a rare but highly lethal cancer. After the identification of gain-of-function mutations in *KIT* and *PDGFRA* receptor genes that opened the way to the treatment with inhibitors of c-KIT and platelet-derived growth factor receptor A (PDGFRA) tyrosine kinase receptors (e.g., imatinib and newer analogs) in succinate dehydrogenase (SDH) competent GIST, it is today known that mutations in other molecules, such as *BRAF* or *RAS*, or mutations in *SDH* subunits that lead to SDH deficient GISTs represent different subtypes with specific features affecting prognosis and clinical outcome. The review of Ravegnini *et al.*^[26]^ focuses on tumor alterations in GISTs in relation to response to the available drugs. A mention of GIST germline DNA alterations and their possible role in drug efficacy/resistance is also provided, although this matter is still the subject of debate.

Liver cancers are substantially unresponsive to cytotoxic anticancer agents. Targeted drugs, in particular antiangiogenic drugs, are today approved for the treatment of hepatocellular carcinoma, in which, however, they are endowed with low efficacy. Alonso-Peña *et al.*^[27]^ reviewed molecular alterations (mainly, germline polymorphisms and somatic mutations) that have been suggested to confer drug resistance to liver cancers (i.e., hepatocellular carcinoma and cholangiocarcinoma). However, despite the availability of a relevant number of studies that report potential associations among polymorphisms/mutations in genes codifying transporters or detoxifying enzymes, changes in molecular targets, enhanced DNA repair mechanisms, altered balance between survival and apoptosis pathways, tumor microenvironment and epithelial-mesenchymal transition, and cytotoxic or targeted anticancer drug efficacy and/or toxicity, no solid data are currently available and further investigation is needed.

Overall, the manuscripts included in this special issue underscore the relevance of germline and somatic DNA variations in response (efficacy and toxicity) to pharmacological treatment in cancer. Examples for which data have been already widely confirmed in several cancer types indicate that personalized oncological treatments represent a fundamental goal to be further pursued in all other malignant conditions and related cancer therapeutics.

## Declarations

### Copyright

© The Author(s) 2020.
